# A dual-loop chemostat to investigate multi-species biofilms on implant surfaces under adjustable flow conditions

**DOI:** 10.3389/fmicb.2026.1751315

**Published:** 2026-02-13

**Authors:** Jan-Ole Reese, Ingrid Maria Castro Lund, Håvard Jostein Haugen, Athanasios Saragliadis, Ståle Petter Lyngstadaas, Dirk Linke

**Affiliations:** 1 Department of Biosciences, University of Oslo, Oslo, Norway; 2 Department of Biomaterials, Institute of Clinical Dentistry, University of Oslo, Oslo, Norway; 3 Corticalis AS, Oslo Science Park, Oslo, Norway

**Keywords:** biofilm model, flow, implant materials, multi-species biofilm, oral biofilm, shear stress

## Abstract

Natural biofilms are typically composed of a mix of different microbial species and are often exposed to strong shear forces resulting from liquid flow. Simple biofilm models that attempt to study biofilms are based on a single species and on static growth conditions. To overcome these limitations, we developed a modular dual-loop reactor that decouples bacterial cultivation from hydrodynamic exposure, enabling independent control of nutrient availability (and thus, cell density) and flow rate (and thus, shear stress). Importantly, the system allows for testing different surface materials in a systematic manner. To validate our setup, we used a community of six keystone members of oral biofilms in conjunction with titanium materials of defined roughness that mimic dental implant surfaces. We found that biofilm mass, robustness, and species distribution not only differ significantly between static and dynamic growth conditions, but also vary strongly with different flow velocities. The biofilms formed under flow could be separated into two fractions, one that washed away very easily, and a more robust, basal layer. At low shear forces, overall biofilm mass was the highest, but at the expense of biofilm robustness. At medium shear forces, the robust fraction of the biofilm had the highest relative content of extracellular matrix. At the highest flow rates, the biofilm mass was low, but late colonizers (represented by the oral pathogens *Porphyromonas gingivalis* and *Aggregatibacter actinomycetemcomitans*) had the lowest relative abundance. This is in accordance with the concept that high flow of saliva reduces the risk of oral disease. Future applications of our system will include the systematic testing of antimicrobial coatings or surface design effects under defined flow regimes, opening the path toward better medical implants.

## 1 Introduction

Biofilms form naturally under flowing conditions that require active surface attachment of microbial cells, in riverbeds and industrial water systems, in biological tissues and on medical devices. Fluid flow plays a crucial role in biofilm development by influencing mass transfer, nutrient diffusion, and local shear gradients within the biofilm matrix. Microstructures that develop under flow conditions, such as wrinkles and channels, aid the mass transfer of solutes and nutrients from the bulk fluid into deeper biofilm layers (Chen et al., 2024). Shear forces generated by fluid flow have been identified as one of the key factors driving structural adaptations in biofilms that cannot be replicated in static conditions (Liu and Tay, 2002). Enhanced shear stress leads to progressively thinner and denser biofilms characterized by a more compact and stable structure (Chang et al., 2020; Kwok et al., 1998; Liu and Tay, 2002; Pechaud et al., 2022; Zhang et al., 2022). Directly correlated is also the stimulated secretion of extracellular polymeric substances (EPS) under shear, benefiting the adhesion and structural integrity of biofilms (Hou et al., 2018; Liu and Tay, 2002). Incorporating flow into *in vitro* biofilm models revealed that biofilm phenotypes and gene expression patterns (particularly those related to virulence or adhesion) are fundamentally altered compared to their statically grown counterparts (Nailis et al., 2010). Given these profound flow-dependent effects on biofilm properties, accurate *in vitro* modeling necessitates the integration of representative hydrodynamic conditions.

Over the past 2 decades, increasingly sophisticated *in vitro* models have been developed that more closely mimic clinically relevant *in vivo* conditions. These provided various systems with distinct advantages and limitations. Static models cultivate bacteria directly in multi-well plates, employing different approaches: Biofilms can be formed on the well surface itself (Sharma et al., 2005), on substrate-discs (e.g., hydroxyapatite) placed on the bottom of the wells (Guggenheim et al., 2001), or on pegs inserted into the wells from above by employing a Calgary Biofilm Device (Ceri et al., 1999). While offering simplicity and high-throughput potential, these static models suffer from nutrient limitation and accumulation of metabolic byproducts (Coenye and Nelis, 2010), cannot incorporate any flow dynamics and often raise the question of whether the cells actively attach to presented surfaces or simply sediment down (Ramachandra et al., 2023).

Semi-dynamic models are primarily drip-fed systems, where growth medium is supplied by dripping it onto a substrate to cultivate biofilms under a thin fluid interface (Abdullah and Jakubovics, 2022). Using a drip-flow biofilm reactor, fluid flow is achieved by simply tilting the system to drain the liquid over the substrate surface (Goeres et al., 2009). In the case of the “constant depth film fermenter” (CDFF), excess biomass is removed by rotating scraper blades that sustain drip-fed biofilms at a uniform thickness (McBain, 2009; Pratten, 2007). While drip-fed systems achieve more consistent biofilm growth by constantly replenishing nutrients, their incorporation of flow, particularly in a controlled manner, remains limited. In contrast, advanced flow-through systems such as flow cells (Palmer and Caldwell, 1995), microfluidic chips (Tang et al., 2022) or microfluidic devices (Benoit et al., 2010) facilitate the simulation of fluid dynamics by precisely controlling flow conditions when passing bacterial culture through small channels. These systems excel at investigating biofilm formation and behavior on a microscopic level, with the benefit of real-time observation (Azeredo et al., 2017). However, their restricted working volumes and channel dimensions render them inadequate for creating entire biofilms on a macroscopic scale and across diverse substrates (Yawata et al., 2016), as often required for a comprehensive assessment of medical applications.

Lastly, the CDC (Centers for Disease Control and Prevention) biofilm reactor and the Modified Robbins device (MRD) represent fully dynamic biofilm systems on a larger scale, both harboring removable coupons or plugs, exposing a surface of interest to continuous culture flow (Abdullah and Jakubovics, 2022). The CDC reactor operates as a stirred-vessel system, where biofilm surfaces are located directly within the cultivation vessel and shear is generated through the rotational stirring motion (An et al., 2022; Rudney et al., 2012). While this design favors the optimization of cultivation conditions to achieve a steady-state culture, the exact flow exposure is difficult to determine, and the non-uniform mixing pattern created by the stirring motion leads to a certain heterogeneity between the different biofilm sites. The MRD, also called flow-over reactor, features removable plugs along a flow channel, presenting the biofilm surfaces into the flow under defined conditions (Azeredo et al., 2017; Luo et al., 2022). While traditional models connected the MRD in-line with bacterial culture or fresh medium (Kharazmi et al., 1999), recent models connected it to the efflux of a continuous culture to provide optimal biofilm growth conditions (Blanc et al., 2014). This configuration comes with a critical limitation: The flow rate within the MRD is directly coupled to the drainage outflow of the bioreactor. This coupling restricts experimental flexibility, as overly high flow rates in the MRD risk a washout of strains from the steady-state culture. At the same time, low flow rates may lead to nutrient starvation within the culture and/or biofilms.

To address these practical limitations in current methodologies, we developed an *in vitro* biofilm model that decouples flow dynamics from cultivation conditions. Our system improves previously reported configurations and enables the independent adaptation of both parameters, leading to a high reproducibility of the resulting biofilms. Employing a dual-cycle approach allows for maintaining precise control of flow velocity over target surfaces within the MRD during biofilm formation. At the same time, in- and outflow of the bioreactor can be individually regulated to achieve desired culture conditions, such as retaining steady-state conditions for optimal growth or inducing nutrient limitation. To demonstrate the capabilities of our decoupled flow-cultivation system, we selected oral biofilms on dental implants as a representative model system that combines the technical challenges of anaerobic multi-species cultivation with clinically relevant flow dynamics. Such relevant dynamic *in vitro* models are crucial to facilitate the development and evaluation of critically needed novel treatments for dental implant infections.

Dental implants have become the standard for tooth replacement in modern dentistry (Buser et al., 2012), improving not only oral health but also patients’ overall quality of life (Johannsen et al., 2012). Concurrently, the increasing prevalence of implant placement has led to a corresponding rise in peri-implantitis (Atieh et al., 2013; Warreth et al., 2015), infections characterized by inflammation and bone loss around osseointegrated implants. The main drivers for these infections are bacterial biofilms, which form on over 40% of dental implants (Algraffee et al., 2012).

These oral biofilms consist of complex, highly structured sessile microbial communities, self-embedded in extracellular polymeric matrix (Marsh, 2005), whose maturation is sequentially organized: Initial adhesion is facilitated by the presence of a pellicle (Hojo et al., 2009). This pellicle is formed by salivary-derived proteins (including mucins), which promote surface attachment of early colonizers such as *Streptococci* and *Actinomyces* (Chahal et al., 2022; Kolenbrander et al., 2002). Serving as “anchors” for subsequent colonization, the latter provide attachment sites for secondary/intermediate colonizers, including *Veillonellae* and *Fusobacteria*. These bridging species support the colonization of further species and form co-aggregates and mutualistic interspecies relationships, creating a metabolic network within the biofilm (Kolenbrander et al., 2010; Zhou et al., 2021). Eventually, late colonizers and potential pathogens, such as *Porphyromonas gingivalis* and *Aggregatibacter actinomycetemcomitans*, can integrate into the mature biofilm (Socransky et al., 1998). While all previous species are common within a healthy periodontal microbiome, the increased incorporation of these periodontal pathogens (dysbiosis) is strongly associated with periodontal disease, as they are a key driver of inflammatory processes (Hajishengallis et al., 2012; Socransky et al., 2002).

Besides their complex multi-species composition, another crucial factor to consider when studying these oral biofilms is their exposure to the highly dynamic environmental conditions within the oral cavity. Oral biofilms are typically challenged by constantly changing hydrodynamic and environmental conditions due to salivary fluid flow, which also transports planktonic bacteria between attachment sites (Hall et al., 2017). Beyond these general factors, the peri-implant environment presents even more complex flow dynamics: Connective tissue fibers orient only parallel to the implant, rather than inserting perpendicularly as they do with natural teeth. The absence of a natural periodontal ligament results in an enlarged sulcular space around the implant (Berglundh and Lindhe, 1996; Emecen-Huja et al., 2013). This sulcular space is flushed with an inflammatory secretion known as peri-implant sulcular fluid (PISF), analogous to gingival crevicular fluid (GCF) around natural teeth (Javed et al., 2011). Both PISF and GCF demonstrate significantly increased volumetric flow rates during inflammatory conditions (Bevilacqua et al., 2016): The flow of crevicular fluid is closely correlated with the severity of periodontal inflammation, serving as a direct indicator of tissue response; it increases markedly due to enhanced vascular permeability and epithelial ulceration at inflamed sites. While healthy gingiva produces minimal GCF, inflammation leads to a substantial rise in both volume and compositional complexity, transforming it into an exudate characteristic of inflammatory processes. In addition to the effects of increased fluid flow on biofilm formation, the associated enhancement in nutrient delivery promotes accelerated plaque accumulation and maturation at inflamed gingival margins (Quirynen et al., 1991). Yamamoto et al. (2021) reported a ∼88% increase in blood flow in the peri-implantitis group compared with natural teeth. Similar increases have been reported in other animal models and other groups (Katz et al., 2025; Mayr et al., 2024; Rodríguez-Martínez et al., 2006; Sugiyama et al., 2012), highlighting the importance of a model system that permits flow rate regulation.

Recognizing and understanding the complex interplay between flow conditions and biofilm growth is crucial for developing critically needed novel therapeutic approaches against peri-implantitis. These are traditionally evaluated in animal studies, whose increasing numbers raise significant ethical concerns, alongside practical limitations regarding cost and scalability (Blanc-Sylvestre et al., 2021; Faggion, 2015; Faggion jr et al., 2016; Rovida, 2009). An alternative is to implement *in vitro* biofilm models that replicate key aspects of the oral environment, simulating bacterial colonization of implant surfaces under controlled laboratory conditions.

When examining biomedical treatments such as debridement applications, mechanically robust biofilms are essential for accurate testing. Weakly attached biofilms would be non-specifically removed during routine washing steps of controls, masking genuine treatment effects. Higher rigidity in biofilms, making them suitable for rigorous experimental testing, can be achieved by applying shear stress during cultivation. For instance, Stoodley et al. (2002) demonstrated that subgingival biofilms formed under high-shear conditions exhibited stronger surface attachment and greater cohesive strength than their low-shear counterparts. This principle guided our approach to using our dynamic *in vitro* model to investigate how varied flow velocities affect the mechanical properties of biofilms on implant surfaces, aiming to identify optimal conditions for debridement testing. The inclusion of six common and representative species from subgingival plaque (Socransky et al., 1998) aimed to reveal the effects of flow on biomass, structural integrity, and species composition in our mixed microbial community.

The central aim of this study was to develop and characterize a dynamic *in vitro* biofilm model that decouples biofilm flow exposure from bioreactor cultivation conditions. The versatility of this system extends beyond the oral biofilm demonstration, which was presented to illustrate the model’s capabilities. An independent control of hydrodynamic and cultivation parameters enables systematic investigation of flow effects across any biofilm-forming community, from single-species industrial biofilms to polymicrobial infections. By replicating physiological flow conditions while optimizing growth conditions to specific microbial consortia, the model is particularly suited to simulate clinical scenarios on various biomaterials: From low-shear orthopedic implants to high-shear urinary catheters, vascular grafts or prosthetic heart valves. Cultivation conditions can also be adjusted to investigate how nutrient availability or environmental factors modulate biofilm properties under flow. This approach shifts biofilm research from the constraints of coupled or in-line systems towards flexible, physiologically relevant *in vitro* models.

## 2 Materials and methods

### 2.1 Bacterial strains and culture conditions

Bacterial strains were selected based on previous publications (Blanc et al., 2014; Hussain et al., 2024) and obtained from the respective labs. Strains used in the study were as follows; *Streptococcus oralis* NCTC 11427, *Actinomyces naeslundii* ATCC 19039, *Veillonella parvula* NCTC 11810, *Fusobacterium nucleatum* DSM 20482, *Porphyromonas gingivalis* ATCC 33277 and *Aggregatibacter actinomycetemcomitans* DSM8324. All strains were initially cultivated on blood agar plates (Blood Agar Base No. 2, VWR Chemicals, Leuven, Belgium), supplemented with 5% (v/v) defibrinated sheep blood (Thermo Scientific Oxoid, Basingstoke, United Kingdom), 5.0 mg L^–1^ hemin (Fisher Scientific, Loughborough, United Kingdom) and 0.5 mg L^–1^ menadione/vit. K_3_ (Sigma-Aldrich, St. Louis, MO, United States). Liquid cultures were grown in modified brain-heart infusion medium (BHI), containing 37 g L^–1^ BHI broth (VWR Chemicals, Leuven, Belgium), 5.0 mg L^–1^ hemin (Fisher Scientific, Loughborough, United Kingdom), 2.5 g L^–1^ mucin (porcine, type II), 1 g L^–1^ yeast extract, 2 g L^–1^ sodium bicarbonate, 0.5 mg L^–1^ menadione/vit. K_3_, 0.5 g L^–1^ cysteine and 0.1 g L^–1^ glutamic acid (Sigma-Aldrich, St. Louis, MO, United States).

To ensure anaerobic conditions, the medium was prepared in a Widdel flask (Laso-Pérez et al., 2018) and dispensed into glass tubes sealed with butyl rubber stoppers using the Hungate technique, as described by Widdel (1980). A gas mixture of N_2_/CO_2_ (95:5, v/v) was used to equilibrate the pH of the bicarbonate-buffered medium to 7.2. All cultures were incubated at 37 °C under anaerobic conditions for 24–72 h.

### 2.2 Continuous culture set-up coupled to flow-controlled biofilm formation

**FIGURE 1:**
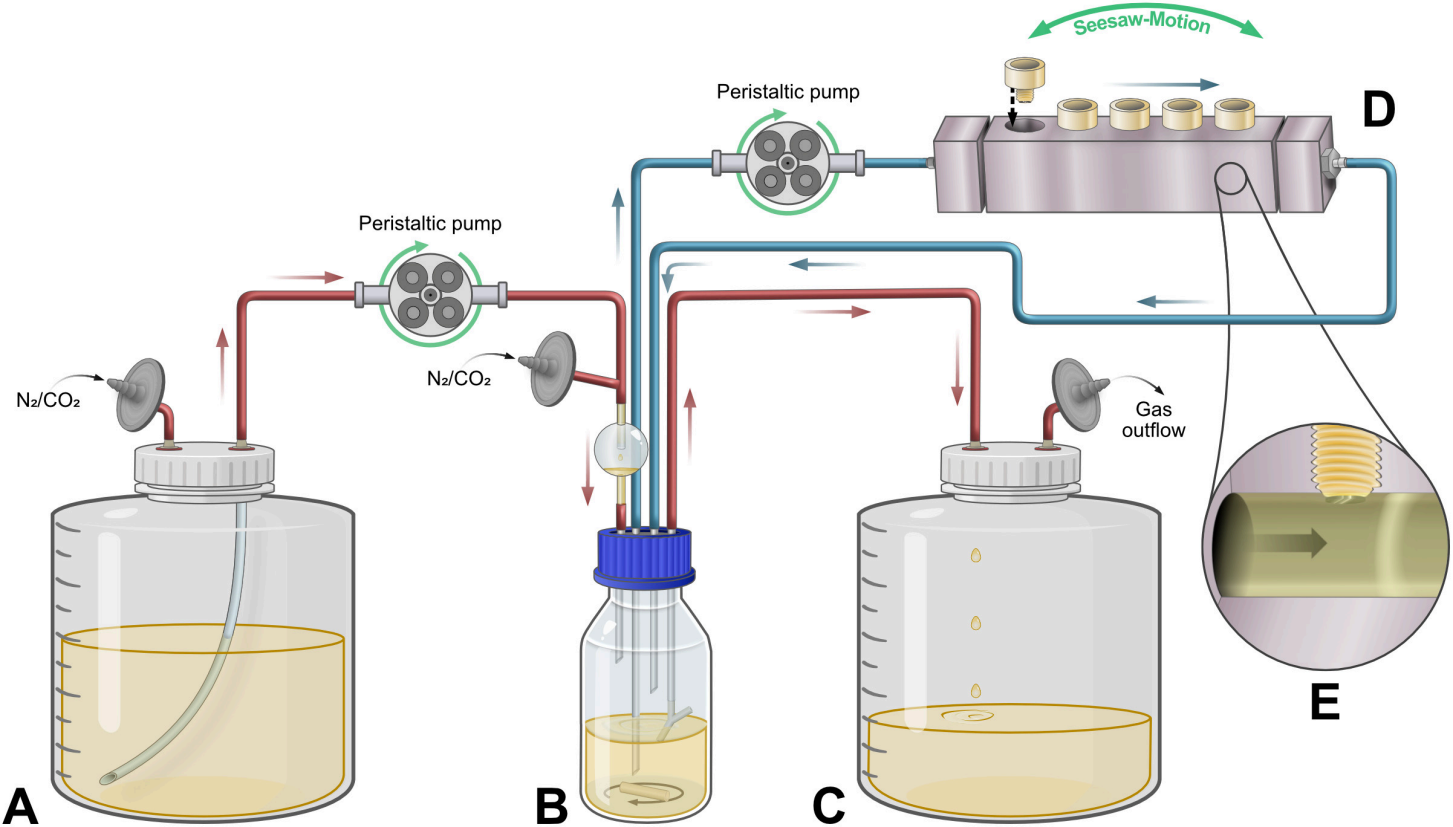
Schematic representation of the flow-controlled biofilm model. Red tubing displays the cultivation cycle, blue tubing the flow-controlled biofilm formation cycle. **(A)** Reservoir medium carboy with sterile filtered gas inlet. **(B)** Culture vessel with magnetically stirred continuous culture. The vessel is placed in a temperature-regulated water bath. **(C)** Waste carboy for culture outflow with gas exhaust. Gas-wash bottle containing 0.5 M NaOH was connected subsequently (not shown). **(D)** Modified Robbins-device (MRD) flow-through chamber with removable sampling plugs holding surface discs for biofilm formation. **(E)** Magnification into the MRD flow-channel cross-section, showing how the sample plugs present only the disk surface to the culture flow.

In order to achieve the independent adjustment of flow condition for biofilm formation and bacterial cultivation, both systems were separated into independent cycles (Figure 1). Cultivation cycle (red): Cells were grown in a 100 mL glass vessel, utilizing a Y-shaped outlet for gas and culture to maintain the culture volume at 50 mL. Excess culture was thus expelled from the system by the gas inflow pressure, once the surface level reached the branching point of the outlet, as described by Cypionka (1986). Reservoir medium and chemostat culture were kept under an atmosphere of N_2_/CO_2_ (95:5, v/v) to maintain anoxic conditions. Fresh medium was fed into the culture vessel at a constant flow rate (*F*), entering combined with the gas inflow through a flow-break to avoid backflow contamination. Steady-state conditions were established by adjusting *F* to set a dilution rate (*D*) of the chemostat that matched the growth rate of the culture (Drake and Brogden, 2014). The continuous culture was incubated at 37 °C in a water bath and stirred magnetically at 350 rpm. A sampling port (Supplementary Figure 1) permitted periodic OD_600_ and pH measurements to confirm steady-state conditions.

Biofilm formation cycle (blue): Two separate sampling ports allowed the independent extraction and refeeding of the culture, without altering the culture conditions. Exact adjustment of flow rate within this cycle was facilitated using a peristaltic pump (Ismatec Reglo Digital Miniflex; Masterflex, United States), leading fresh culture through a Modified Robbins Device (MRD). The MRD is an artificial multiport sampling flow-through chamber, allowing continuous flow of culture over removable sampling plugs (Figure 1E), which hold coins that present the surface of interest for biofilm formation (Kharazmi et al., 1999).

Based on the cross-sectional area (*A*) of 1.21 cm^2^ in the flow channel of our MRD (MPMR-10PSF, Tyler Research Corp., Alberta, Canada) and the volumetric flow rate (*Q*) set by the peristaltic pump, the exact flow velocity ($\vec{\text{v}}$) to which the biofilms are exposed was calculated:


$$\vec{v}=\frac{Q\,\left(a\,d\,j\,u\,s\,t\,a\,b\,l\,e\,f\,l\,o\,w\,r\,a\,t\,e\right)}{A\,\left(1.21\,c\,m^{2}\right)}$$

The Reynolds number (Re) for the flow within the modified Robbins device was estimated to characterize the hydrodynamic regime, assuming water-like properties of the growth medium at 37 °C (density ρ = 997 kg m^–3^, dynamic viscosity μ = 0.0007 Pa⋅s) and a hydraulic diameter of 12.4 mm, corresponding to a channel cross-sectional area of 1.21 cm^2^. Flow velocities of 8, 16, and 32 cm min^–1^ (0.00133–0.00533 m s^–1^) yielded Reynolds numbers of approximately 24, 47, and 94, respectively. These values indicate fully laminar flow conditions in the Robbins device. The Reynolds number (Re) was calculated to characterize the flow regime within the Robbins device using the following relation:


$$R\,e=\frac{\rho \,\mathrm{vD}}{\mu }$$

where: Re = Reynolds number (dimensionless), ρ = fluid density (kg⋅m^–3^), v = average flow velocity (m⋅s^–1^), D = hydraulic diameter of the flow channel (m), and μ = dynamic viscosity of the fluid (Pa⋅s).

Incubation of biofilms at 37 °C was facilitated by placing the MRD within a separate incubator. The device was mounted on a rocker shaker (Thermo Fisher Scientific), slowly tilting the MRD in a seesaw motion to ± 15° at 1 rpm. This allowed gas bubbles, formed by bacterial gas production, to escape the system while preventing the deposition of cell debris in the flow channel, especially at lower flow rates. The total working volume of the cultivation system was 100 mL, which included both the culture vessel and the recirculating components (tubing and MRD) of the continuous flow setup.

### 2.3 Preparation of titanium surfaces

To emulate peri-implantitis infections in the biofilm model, the sampling plugs (*n* = 10) of the MRD were equipped with studs of grade II titanium, presenting a disc surface area of 50 mm^2^. Titanium surfaces were modified to resemble the rough features of dental implants, characteristic of commercial OsseoSpeed^®^ surfaces (Dentsply Sirona, Charlotte, United States). These were produced according to a previously described procedure (De Lauretis et al., 2025b). In short, stud surfaces were grit-blasted and sequentially washed with (1) 40% v/v NaOH, (2) 50% v/v HNO_3_, and (3) MilliQ water in an ultrasonic bath; upon reaching a neutral pH, the surfaces were acid-etched in 0.2% v/v HF at pH 2.1 for 2 min. The treated surfaces were stored at room temperature in EtOH until utilization. The treated stud surface exhibited moderate roughness, with a Sa of approximately 2.1 μm effective fluid retention in the core region (Sci ≈ 1.5), negative skewness (Ssk ≈−0.3) and high kurtosis (Sku ≈ 4) (De Lauretis et al., 2025a; Wang et al., 2025).

### 2.4 Preparation of dynamic multi-species oral biofilms

The modified BHI medium described earlier was used as a growth medium for the cultivation of a multi-species oral community within the chemostat model. Anoxic conditions were created by autoclaving the medium directly in the reservoir carboy and flushing the headspace with N_2_/CO_2_ (95:5, v/v), while still hot. Potentially remaining oxygen traces were reduced by the subsequent addition of cysteine as described above. The whole system was then filled with the growth medium, while constantly kept under an anoxic atmosphere, and operated overnight to ensure sterility and facilitate anoxic conditions. The CO_2_ concentration of 5% in the system atmosphere kept a pH of 7.2 in the bicarbonate-buffered medium. The flow rate of the peristaltic pump, leading medium/culture through the MRD in the biofilm cycle, was adjusted independently for each run to flow velocities of 8–40 cm⋅min^–1^.

A pooled bacterial inoculum was prepared by combining pre-cultures of all six bacterial strains. Individual strains were grown under anaerobic conditions as described above until mid-exponential phase. Strain ratios of the inoculum were adjusted to create a starting culture in the bioreactor, consisting of 10^3^ colony-forming units (CFU)/mL of *S. oralis*, 10^4^ CFU/mL of *V. parvula* and *A. naeslundii*, 10^5^ CFU/mL of *F. nucleatum* and 10^6^ CFU/mL of *A. actinomycetemcomitans* and *P. gingivalis*. The system was initially operated in batch mode for 12 h, to allow the culture to establish, while determining the doubling time by regular measurements of optical density (OD_600_). A reactor residence time was chosen accordingly, matching the doubling time to ensure that the culture is neither washed out by too high, nor starved by too low medium turnover. Flow rate of the growth medium feed was therefore calculated, according to:


$$F\,l\,o\,w\,r\,a\,t\,e\,\left(m\,L*m\,i\,n^{-1}\right)=\frac{R\,e\,a\,c\,t\,o\,r\,s\,y\,s\,t\,e\,m\,v\,o\,l\,u\,m\,e\,\left(100\,m\,L\right)}{R\,e\,s\,i\,d\,e\,n\,c\,e\,t\,i\,m\,e\,\left(m\,i\,n\right)}$$

The reactor system was then incubated in continuous culture for 72 h. Subsequently, biofilms formed on the titanium stubs in the MRD were removed and gently rinsed in sterile Ringer’s solution (100 mM NaCl, 4 mM KCl, 2 mM CaCl_2_⋅2H_2_O, 1 mM MgCl_2_⋅6H_2_O, 30 mM Na(CH_3_COO)⋅3H_2_O; pH 7.0) to remove loosely attached cells. Harvested biofilms were either processed immediately for downstream analysis or stored at −20 °C for subsequent DNA extraction.

### 2.5 Preparation of static multi-species oral biofilms

Biofilms were prepared under static conditions to provide an additional reference: Grade II titanium discs with a surface area of 30 mm^2^, prepared analogous to the MRD studs described above, were placed within the wells of a non-treated, polystyrene 24-well tissue culture plate (VWR International, Leuven, Belgium). Wells were filled with 1.5 mL mixed bacterial culture matching the initial bioreactor inoculum and incubated under an anoxic atmosphere of N_2_/CO_2_ (95:5, v/v) for 84 h. Processing of static biofilm discs was conducted analogously to MRD studs.

### 2.6 Assessment of mechanical biofilm resistance by standardized washing procedure

A portion of all sampled biofilms was immediately assessed for structural integrity and surface adhesion strength by subjecting the samples to a standardized washing procedure (marked as “washed” biofilms). In contrast to biofilms marked as “untreated,” these biofilms were immediately after sampling exposed to five consecutive rinses with 1 mL sterile NaCl (0.9%). The washing fluid was directly applied to the biofilm surface in a uniform pattern and strength.

### 2.7 Total biomass determination

To measure the total biofilm biomass, samples were first air-dried at 37 °C for 15 min to stabilize the structure and prevent detachment during subsequent processing. The biomass adhering to the titanium surface was then stained with 0.1% crystal violet (CV) solution for 30 min. Following staining, the stubs were washed four times with 2 mL of Ringer’s solution to remove excess unbound dye. The bound CV was then extracted from the biofilms using 96% ethanol, aided by rigorous vortexing and ultrasonication. Absorbance of the solubilized CV, correlating to biofilm biomass, was measured at 590 nm using an Epoch 2 microplate spectrophotometer (Agilent BioTek, VT, United States). Titanium stubs incubated in sterile modified BHI medium were processed identically and used as blank/background controls.

### 2.8 DNA extraction

Genomic DNA was extracted from biofilms using a modified phenol-chloroform method optimized for mixed microbial populations on titanium surfaces. To ensure complete DNA recovery from all cells, including those firmly adhered to the titanium surface, lysis was performed directly on the titanium stubs. Briefly, biofilms were subjected to three freeze/thaw cycles followed by enzymatic lysis in buffer containing 20 mM Tris⋅HCl (pH 8.0), 2 mM EDTA, 1.2% Triton X-100, 0.1% Tween-20, and 20 mg/mL lysozyme. Biofilm detachment was facilitated by vigorous vortexing, followed by ultrasonication (20 min, 37 °C), and then incubation at 37 °C for an additional 30 min to complete cell lysis. Protein digestion was performed using Proteinase K (20 mg/mL) in complete lysis buffer (100 mM Tris-HCl, pH 8.0, 20 mM EDTA, 200 mM NaCl, 2% SDS) at 56 °C for 1 h.

DNA was extracted from the lysate by mixing with an equal volume of phenol:chloroform:isoamyl alcohol (25:24:1; saturated with 10 mM Tris, 1 mM EDTA, pH 8.0) and separating the phases by centrifugation (16,000 × g, 5 min). The aqueous phase was reextracted accordingly with one volume chloroform:isoamyl alcohol (24:1) to remove potential phenol contaminations. Precipitation of DNA was performed by adding 1/10 volume of 3 M sodium acetate, 1 μL of glycogen (20 μg/μL), and 2.5 volumes of ice-cold absolute ethanol, followed by overnight incubation at −20 °C. The precipitated DNA was collected by centrifugation (16,000 × g, 30 min, 4 °C), washed twice with 70% ethanol, air-dried, and resuspended in 50 μL of 10 mM Tris-HCl (pH 8.0). Concentration and purity were assessed using a NanoDrop ND-1000 spectrophotometer (Thermo Fisher Scientific, DE, United States).

### 2.9 Species-specific quantification by quantitative PCR

Quantitative PCR was employed to determine the abundance of each bacterial species within the multi-species biofilms. A novel highly species-specific primer set was designed for all six strains used in this study, targeting the RNA polymerase beta-subunit gene (*rpoB*). This target gene was selected since it offers several advantages, compared to the traditionally targeted multi-copy 16S rRNA gene: As a single-copy housekeeping gene, *rpoB* prevents the overestimation of species abundance while concurrently offering a better resolution at the species level (Adékambi et al., 2009; Ogier et al., 2019).

All primer pairs, as listed in Table 1, were designed to have similar yet distinctive amplicon lengths (114–198 bp) and compatible annealing temperatures (∼65 °C) to enable simultaneous amplification in a single qPCR run. Primer specificity was confirmed by BLAST analysis against the NCBI database, and potential off-target amplification was experimentally evaluated against genomic DNA from each species in the biofilm model to ensure specificity in a mixed community.

**TABLE 1:** Newly developed qPCR primers used in this study.

Target species	Strain ID	Gene	Primer sequence	Amplicon size (bp)
*S. oralis*	NCTC 11427	*rpoB*	F-ACAGGCGAAATCAAGACCCAA R-GCGGACCAACTGAGAAACG	114
*A. naeslundii*	ATCC 19039	*rpoB*	F-GTACCCACCGACACCATCTG R-CGACGAGACCCACTACCTGA	198
*V. parvula*	NCTC 11810	*rpoB*	F-TGAGTGGTTCTTGGAGAGTGG R-CGAAGTGGTGCTGCGTAAG	168
*F. nucleatum*	DSM 20482	*rpoB*	F-TCAACAACAACTCCTTTAGAACCA R-GAGAAACAGAACCACCTGCTG	118
*P. gingivalis*	ATCC 33277	*rpoB*	F-TGGGGAGATAGTAGGTGACGA R-CAGATTGTGGCAGAGAGGGG	130
*A. actinomycetemcomitans*	DSM 8324	*rpoB*	F-TTTCACATCGGAGGCTTTTTCAC R-TATCGTGTATATCGGTGCGGAAG	136

qPCR reactions were performed with a total volume of 10 μL, containing 5 μL of SYBR qPCR Master Mix (VWR, Leuven, Belgium), 0.25 μL of 10 μM forward/reverse primer (final concentration per reaction: 0.25 μM, each), 2 μL of template DNA, 2% dimethyl sulfoxide (DMSO) and rest nuclease-free water. Amplification was carried out in a LightCycler^®^ 96 System (Roche, Switzerland) with the following thermal cycling conditions: Initial denaturation at 95 °C for 15 min, followed by 40 cycles of denaturation at 95 °C for 15 s and annealing/extension at 66 °C for 30 s with fluorescence acquisition. A melt curve analysis was performed after each run (66–95 °C with 0.2 °C increments). All samples were run in triplicates and data analysis was carried out using the LightCycler^®^ 96 Application Software (V1.1, Roche Diagnostics).

Standard curves for species quantification were generated from pure cultures of all individual species, and the performance of qPCR primers-pairs was assessed as described by Knutsson et al. (2002). For each species, 10-fold dilutions of DNA with known concentration were prepared within the range of 10 ng – 1 pg total DNA per reaction. Generating a standard curve of these dilutions against the resulting quantification cycle (C_*q*_) values, to acquire the slope of the log-linear range (*s*), allows to calculate the amplification efficiency (*E*) for each primer pair (Klein et al., 1999):


$$E=10^{-1/s}-1$$

Since all strains harbor only a single copy of the targeted *rpoB* gene, relative abundances of species within the mixed biofilms were determined as percentage of g DNA relative to the total detected DNA amount in each sample.

### 2.10 Statistical analysis

Data plotting and statistical analysis were performed using the software Origin2022b (OriginLab Corp., MA, United States) and StataSE 17 (StataCorp, TX, United States). Each flow rate was evaluated in two separate bioreactor runs (biological replicates), each containing ten individual titanium surfaces (technical replicates). The sample size (*n*) for each group (Untreated/Washed) is indicated alongside the respective results. Initially, normal distribution of datapoints within the groups (Untreated/Washed) was assessed by Shapiro-Wilk test. Significance levels within groups were determined either by one-way ANOVA for normally distributed data, or by Kruskal-Wallis test for non-normally distributed data. Pairwise comparisons between flow rates within each group were conducted using two-sample *t*-test for normally distributed data, or Mann-Whitney U test for non-normally distributed data. Statistical significance was set at *p* < 0.05.

## 3 Results

A six-species bacterial consortium was selected for the *in vitro* model, representing each stage of the typical colonization sequence in natural oral biofilms and implant-related infections: The early colonizers *Streptococcus oralis* and *Actinomyces naeslundii* were incorporated to facilitate initial adhesion to the implant-like titanium surface. As representative middle colonizers, *Veillonella parvula* and *Fusobacterium nucleatum* were chosen to establish a bridging network that supports further bacterial recruitment and inter-species metabolic relationships within the developing biofilm. The consortium was completed with the late colonizers and periodontal pathogens *Porphyromonas gingivalis* and *Aggregatibacter actinomycetemcomitans*, which integrate into mature biofilm structures and drive inflammatory responses during *in vivo* peri-implant infections. This consortium has been described previously by Sánchez et al. (2011) and Blanc et al. (2014) as an appropriate species selection to produce representative biofilms under both, static and dynamic conditions.

For the reactor-inoculum, species ratios were adapted from Blanc et al. (2014). However, species concentrations were proportionally increased to achieve an initial OD_600_ of 0.1 in the reactor culture. This elevated inoculum density was implemented to prevent species washout during culture establishment, accounting for the short reactor residence time required to maintain the mixed culture under optimal continuous growth conditions. All selected strains demonstrated successful co-cultivation in the reactor culture and formed stable mixed-species biofilms on titanium surfaces, with species diversity maintained throughout cultivation as confirmed by qPCR detection of all consortium members (see section 3.3).

### 3.1 Optimization of qPCR setup and performance

Initial qPCR experiments revealed that samples containing *A. naeslundii* DNA presented an elevated baseline fluorescence prior to amplification, with values of approximately double those of the five remaining strains (Supplementary Figure 2). Analysis of a 5-fold dilution series revealed that this initial baseline fluorescence increased proportionally with *A. naeslundii* template concentration (Supplementary Figure 3A). While low DNA concentrations of 0.2 ng/μL presented no elevation, high DNA concentrations (25 ng/μL) produced baseline fluorescence approximately four times higher than reference measurements. Consequently, qPCR analysis of *A. naeslundii* DNA at higher concentrations showed a delayed progression to the amplification threshold, incorrect assignment of C_*q*_ values, and reduced reproducibility between replicates (Supplementary Figure 3B).

Initially, we suspected that impurities were the origin of this background fluorescence. Switching the DNA extraction method from commercial extraction kits to the phenol-based method presented in this study improved the DNA purity (based on 260/280 and 260/230 ratios). Yet the background fluorescence issue persisted, ruling out contaminants as the cause. Hence, it was concluded that the cause must involve the nucleic acid material itself. Although this specific issue has not been described so far for *A. naeslundii* qPCR analysis, this species is known for a characteristically high GC content (Morou-Bermudez and Burne, 1999), which increases resistance to initial denaturation and promotes formation of secondary structures (Gibson, 2022). In order to prevent the presence of double-stranded regions or hairpins, potentially binding SYBR dye in the early qPCR phase, the preincubation for DNA denaturation (95 °C) was extended to 15 min.

Further, the addition of various concentrations of betaine and DMSO was examined (Supplementary Figure 4), as these have been shown to improve qPCR performance of GC-rich templates (Henke et al., 1997; Shammas et al., 2001). While betaine annihilated fluorescence baseline elevation at all tested concentrations, it also delayed template amplification and compromised inter-replicate consistency. Conversely, DMSO addition improved both template amplification and reproducibility, while successfully counteracting increased baseline fluorescence. The optimal concentration was determined to be at 2% DMSO, which reduced baseline fluorescence to levels corresponding to reference measurements of the other species, while concurrently achieving the lowest C_*q*_ and best reproducibility (Supplementary Figure 4B). After experimentally confirming that 2% DMSO does not impair the amplification of the remaining five target strains used in this study, this concentration was incorporated into the MasterMix for all subsequent qPCR measurements.

In order to find the ideal common annealing temperature for all six primer-pairs, a gradient qPCR was carried out, assessing annealing temperatures between 62 and 69 °C. The optimal annealing temperature was established at 66 °C, which allows for specific annealing and the absence of dimers while ensuring uninhibited amplification (Supplementary Figure 5). Standard calibration curves with genomic DNA demonstrated linear dynamic ranges between 10^–5^ and 10^–9^ mg DNA for all strains (Supplementary Figure 6). All standard curves exhibited strong linearity, with correlation coefficients (*R*^2^) of ≥ 0.998. As shown in Table 2, qPCR amplification efficiencies ranged between 97 and 102% for most primer pairs, with the exceptions of *V. parvula* and *F. nucleatum*, reaching efficiencies of 92 and 81%, respectively. Although the *F. nucleatum* showed a slightly lower efficiency, this is still within the acceptable range for quantitative qPCR applications (Hermann-Bank et al., 2013; Nybo, 2011). Further, the use of species-specific standard curves compensates for efficiency differences, enabling accurate conversion of C_*q*_ values to DNA concentrations for all species.

**TABLE 2:** qPCR primer performance.

Target species	*R* ^2^	*E*
*S. oralis*	0.999	1.019
*A. naeslundii*	0.999	0.980
*V. parvula*	0.999	0.918
*F. nucleatum*	0.998	0.805
*P. gingivalis*	0.999	0.974
*A. actinomycetemcomitans*	0.999	0.999

*E* specifies amplification efficiency, calculated with the slope (s) of the regression line between log (g DNA) and corresponding C_q_, according to *E* = 10^–1/s^ −1. *R*^2^ displays the respective regression coefficient.

### 3.2 Flow velocity shapes biofilm formation and cohesion

**FIGURE 2:**
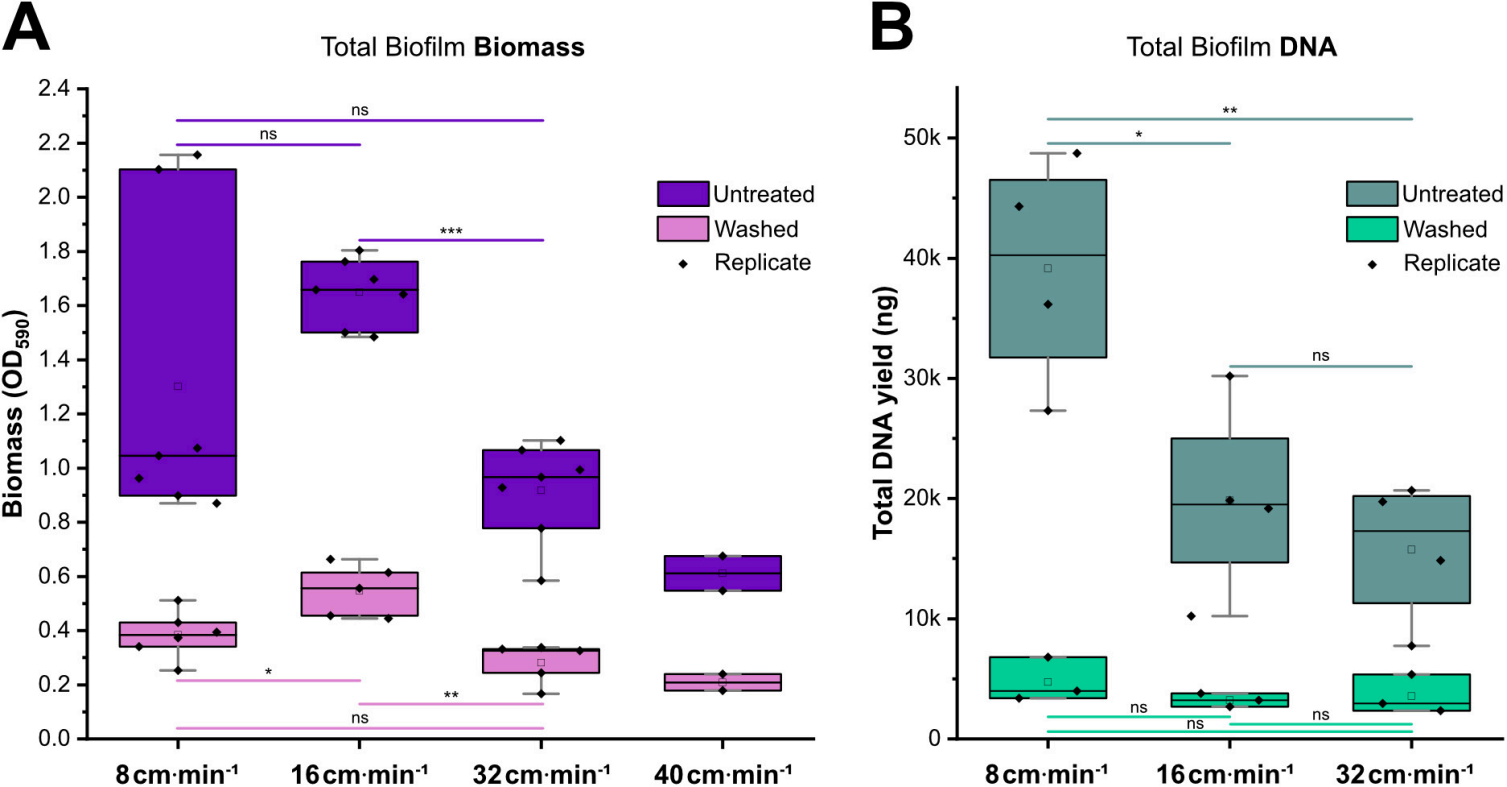
Flow-dependent formation of biofilm biomass and structural integrity. **(A)** Biomass quantification by crystal violet assay (CV) of untreated (*n* = 7) and washed biofilms (*n* = 5). Biomass-bound CV was quantified photometrically at λ = 590 nm. **(B)** Total DNA yield, extracted from untreated (*n* = 4) and washed biofilms (*n* = 3). Nucleic acids were extracted by chemical lysis and phenol:chloroform isolation. Concentration and purity were determined by NanoDrop. Biofilms were grown for 84 h on Ti-stubs in a Robbins device under varying flow velocities ($\vec{\text{v}}$). Washing procedure consisted of five consecutive rinses with 1 mL physiological saline to assess biofilm cohesion. Box plots show median (line), quartiles, mean (□) and individual replicates (◆). Data represents two independent runs for each $\vec{\text{v}}$, with the exception of 40 cm ⋅ min^–1^, which was excluded from further replicates, statistical analysis and DNA extraction, due to insufficient biofilm formation. Statistical differences between flow rates were assessed by Mann-Whitney U test for untreated biofilm biomass samples, due to non-normally distributed data within the 8 cm ⋅ min^–1^ flow rate. All remaining samples were compared by Two-sample *t*-test. Significance: ***p < 0.001, **p < 0.01, *p < 0.05, ns, not significant.

The impact of varying flow velocities, and thus shear stress, on biofilm formation and integrity was assessed by precisely controlling the flow over the model biofilms without changing overall culture conditions, utilizing the capability of our dual-flow system. Six-species oral biofilms were cultivated on implant-like titanium surfaces under flow velocities ($\vec{\text{v}}$) ranging from 8 to 40 cm ⋅ min^–1^. Visual inspection revealed thicker, fluffy biofilms at the lowest tested flow velocity (8 cm ⋅ min^–1^), while the biofilms became more condensed with increasing flow exposure. Biofilm biomass was examined by crystal violet staining before and after a standardized washing procedure, to also assess structural integrity (Figure 2A). The untreated biofilms cultivated under the lowest $\vec{\text{v}}$ (8 cm ⋅ min^–1^) presented a high variance between replicates and therefore no significant difference to the other $\vec{\text{v}}$. This likely reflects the inherently loose structure of these low-flow biofilms, which were susceptible to partial detachment during the washing procedures within the crystal violet staining protocol (required to remove unbound dye). Biofilms grown under moderate $\vec{\text{v}}$ (16 cm ⋅ min^–1^) reached the highest median biomass, which decreased significantly with increasing $\vec{\text{v}}$. Mechanical dislocation of weakly attached biomass by standardized washing revealed a persistent basal layer in biofilms of all groups. While the biomass of this basal layer was largely equal across all flow conditions, biofilms cultivated under moderate $\vec{\text{v}}$ (16 cm ⋅ min^–1^) retained significantly higher median biomass compared to all other tested $\vec{\text{v}}$.

Total DNA content of the biofilms was extracted and quantified (Figure 2B), providing an additional parameter that allows for subsequent determination of species abundances. Since cell-free organic matter detected by the CV-assay (such as EPS) is not taken into account with this method, total DNA content was used as an alternative and potentially more precise indicator of bacterial biomass. The accuracy of the DNA content was refined by implementing a tailored DNA extraction protocol, optimized to maximize DNA recovery from rough implant surfaces. Contrasting the total biomass determination, the lowest $\vec{\text{v}}$ of 8 cm ⋅ min^–1^ presented the highest DNA content, significantly superior to all other flow conditions. Inter-replicate variance was reduced, compared to the biomass assay, likely due to reduced losses of cell material during processing with this method. No statistically significant differences were detected between the moderate (16 cm ⋅ min^–1^) and high flow conditions (32 cm ⋅ min^–1^). Analogous to the total biomass measurements, standardized washing revealed the presence of a robust basal layer in biofilms from all tested flow conditions. The total DNA content of this persistent basal layer remained statistically equivalent across all tested flow velocity conditions.

Measurement of total biomass from static control biofilms was not feasible, since obtained biofilms were too loosely attached to withstand the necessary washing procedures of the CV-assay. Total DNA content of static biofilms (Table 3) was substantially lower compared to biofilms cultivated in the dynamic model. It must be noted that the titanium discs examined in the static model had a slightly smaller surface area than those used in the dynamic model. Standardized washing of static biofilms also resulted in complete biofilm removal and was hence excluded from further analysis. In addition, viability assessment of biofilms was compromised by oxygen exposure during sampling. This resulted in a progressive decline of recovered CFU from sequentially sampled replicate biofilms in preliminary experiments (data not shown), likely due to the death of the obligate anaerobe species fraction.

**TABLE 3:** Relative species abundance (%)—static vs. dynamic biofilms.

Biofilm conditions	Static	8 cm ⋅ min^–1^	16 cm ⋅ min^–1^	32 cm ⋅ min^–1^
*S. oralis*	44.0%	0.67%	21.9%	4.07%
*A. naeslundii*	14.8%	1.45%	26.3%	9.34%
*V. parvula*	14.4%	22.2%	29.6%	44.6%
*F. nucleatum*	20.7%	62.7%	19.4%	41.9%
*P. gingivalis*	3.63%	4.82%	0.30%	0.006%
*A. actinomycetemcomitans*	2.43%	8.16%	2.41%	0.009%
Total biofilm DNA	0.60⋅10^4^ ng ± 0.006⋅10^4^ ng	3.91⋅10^4^ ng ± 0.47⋅10^4^ ng	1.99⋅10^4^ ng ± 0.41⋅10^4^ ng	1.58⋅10^4^ ng ± 0.30⋅10^4^ ng

Total biofilm DNA: average values + standard error of mean (SEM). Relative species abundance: data only contains untreated biofilm samples.

### 3.3 Comparison of biofilm community and relative abundance at varying flow conditions

**FIGURE 3:**
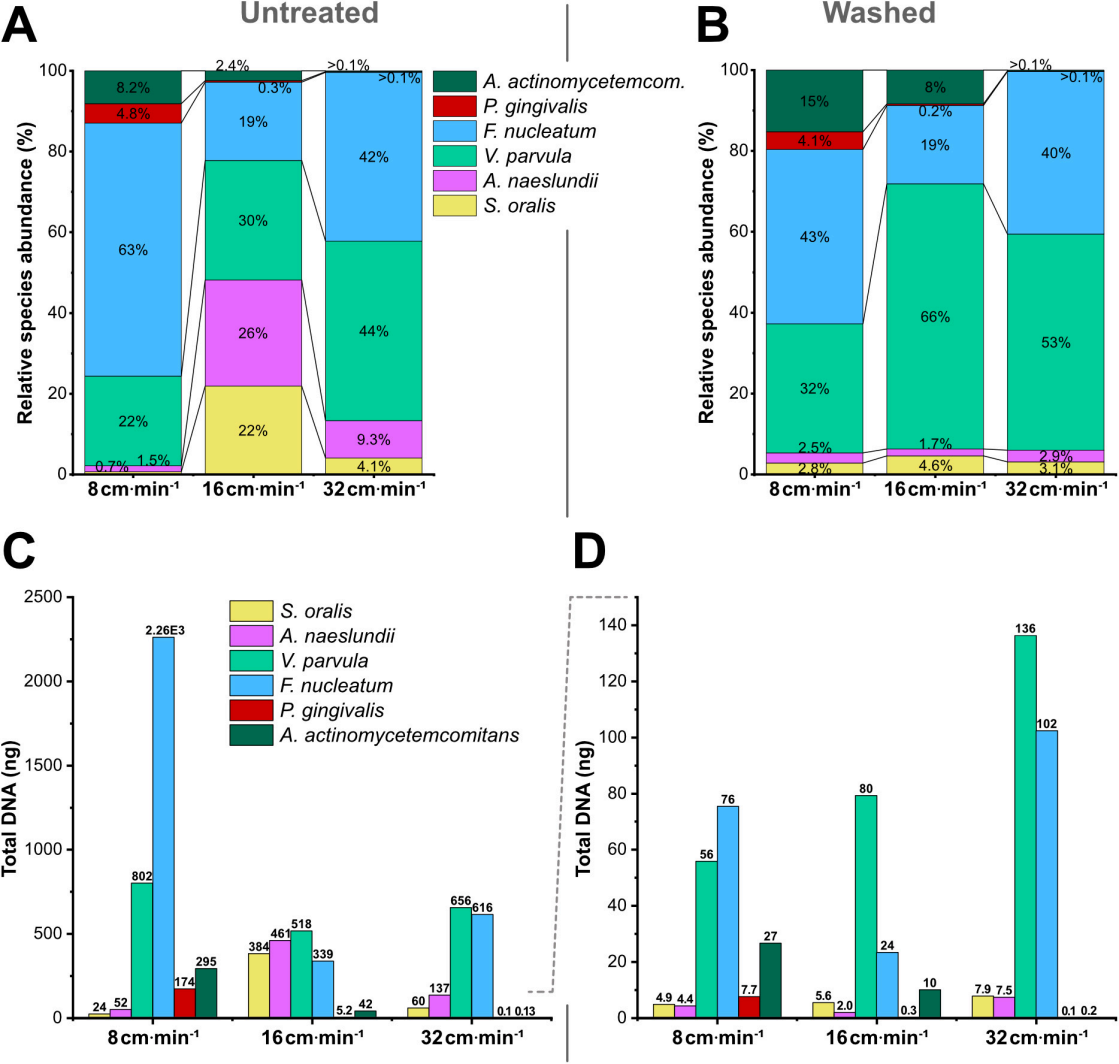
Flow velocity-dependent shifts in microbial community composition of biofilms. **(A,B)** Relative species abundance (%) in untreated **(A)** and washed **(B)** biofilms, cultivated under varying flow velocities. **(C,D)** Absolute quantification of total DNA content per species in untreated **(C)** and washed **(D)** biofilms. Dotted line indicates diminished y-axis scale. Six-species biofilms were grown for 84 h on Ti-stubs in a Robbins device under varying flow velocities ($\vec{\text{v}}$). Washing procedure consisted of five consecutive rinses with 1 mL physiological saline to assess biofilm cohesion. Species quantification of biofilm DNA was performed by SYBR-based qPCR with species-specific primers targeting the single-copy *rpoB* gene.

To investigate the effect of flow conditions on the species composition within the biofilm model, species-specific qPCR was performed (Figure 3). As quantification target, the *rpoB* gene was chosen due to its single-copy nature: With only one copy present per bacterial cell, strains could be directly compared, and DNA quantification directly translated to cell abundance.

Examining untreated *in vitro* biofilms (Figures 3A, C) revealed distinct community shifts in response to changing flow exposure: At the lowest tested $\vec{\text{v}}$ (8 cm ⋅ min^–1^), the biofilm composition was largely dominated by *F. nucleatum*, comprising 63% of the total microbial population, followed by the other middle colonizer *V. parvula*, with 22%. Notably, early colonizers *S. oralis* and *A. naeslundii* represented minimal fractions (0.7 and 1.5%, respectively) and showed the lowest total abundances among all tested flow conditions (Figure 3C). In contrast, late colonizers *P. gingivalis* (4.8%) and *A. actinomycetemcomitans* (8.2%) reached their highest relative and total abundances under these low-flow conditions.

Moderate $\vec{\text{v}}$ (16 cm ⋅ min^–1^) promoted a more evenly distributed biofilm community, with comparable relative abundances between 19 and 30% for all early- and middle colonizers. Conversely, late colonizer abundance of *P. gingivalis* and *A. actinomycetemcomitans* decreased to 0.3 and 2.4%, respectively.

High $\vec{\text{v}}$ conditions (32 cm ⋅ min^–1^) resulted in a middle colonizer co-dominance, with *F. nucleatum* and *V. parvula* each comprising approximately 40% of the community. This shift was primarily driven by a suppression of the total abundance of early colonizers, resulting in relative abundances of 4.1% for *S. oralis* and 9.3% for *A. naeslundii*. Additionally, both late colonizers decreased in relative abundance to values > 0.1%.

Quantification of biofilms after standardized washing was consistent with the previous observation of a persistent uniform basal layer. The bacterial load of washed biofilms remained within a comparable order of magnitude, regardless of cultivation $\vec{\text{v}}$ (Figure 3D). Species distribution presented a uniform pattern across all tested $\vec{\text{v}}$ conditions: Early colonizers maintained a combined relative abundance of ∼ 5% in all flow groups (Figure 3B). The middle colonizers continued to dominate the community composition, with their combined relative abundance progressively increasing from 75% under low $\vec{\text{v}}$, to 85% under moderate $\vec{\text{v}}$ and 93% under high $\vec{\text{v}}$ conditions. Thereby, the total abundance of *V. parvula* increased proportionally with flow exposure, while *F. nucleatum* displayed reduced total abundance under moderate $\vec{\text{v}}$ conditions (16 cm ⋅ min^–1^) compared to the other flow groups. Conversely, the combined abundance of both late colonizers demonstrated flow-dependent reduction and decreased from ∼19% under low $\vec{\text{v}}$, to ∼8% under moderate $\vec{\text{v}}$ and > 0.1% under high $\vec{\text{v}}$ exposure.

Comparison of the (untreated) dynamic biofilms, grown under flow exposure, to reference biofilms, cultivated under static conditions in the absence of flow, revealed how flow fundamentally restructures the community composition (Table 3). Static biofilms exhibited a clear dominance of early colonizers, with *S. oralis* and *A. naeslundii* representing 44% and ∼15% of the population, respectively. This is in strong contrast with the middle colonizer dominance in dynamic biofilms, where early colonizers presented only a small fraction in most biofilms. Late colonizers represented the smallest fraction in static biofilms, reaching with combined ∼5% a share comparable to dynamic biofilms (Supplementary Figure 7).

## 4 Discussion

In the present study, we describe the successful development and validation of a dual-cycle biofilm cultivation system to independently control flow dynamics and cultivation parameters for *in vitro* biofilms. This approach addresses a fundamental challenge in biofilm research: Due to the recognized importance of fluid flow for the representative imitation of natural biofilm behavior in *in vitro* models, the state-of-the-art has moved away from purely static models to dynamic systems. Yet existing models compromise between either precise flow exposure of biofilms or optimal regulation of culture conditions. The here presented model overcomes these constraints by decoupling the biofilm formation under defined flow parameters in a MRD from the cultivation of a microbial consortium in a small-scale bioreactor under steady-state conditions. Reducing the reactor volume to a minimum while simultaneously recirculating the MRD flow through back into the system brings the additional advantage of minimizing the required medium throughput for long-term experiments. The power of this *in vitro* model was demonstrated by cultivating six-species oral biofilms, which emulated peri-implant infections, under low-, moderate- and high-shear conditions. The calculated Reynolds numbers (Re ≈ 20–100) fall within the laminar regime, consistent with physiological flow conditions in the gingival crevice and peri-implant sulcus, where viscous forces dominate fluid movement, reflecting the hydrodynamic conditions relevant for oral implant surfaces (Krastev and Filipov, 2020 for detailed flow rate estimation see Supplementary Material 1). By examining produced biofilms regarding their biomass, nucleic acid content and species composition by qPCR, we could demonstrate that flow velocity distinctly shapes biofilm architecture, robustness and microbial community patterns.

Variation in flow velocities exerted a profound influence across multiple dimensions of the biofilms created with our model. Total biomass exhibited a maximum at low to moderate flow velocities (8–16 cm ⋅ min^–1^) and then decreased significantly with increasing flow. Comparable results were demonstrated by Nairn et al. (2024) in a multi-well plate model, where applied shear stress promoted adhesion and biofilm accumulation of single-species *S. gordonii* and *ex vivo* plaque biofilms until an optimal threshold, above which further shear stress reduced total biomass.

The observed discrepancy between total biomass (measured with a crystal-violet-based assay) and cellular biomass (determined by DNA content quantification) at moderate flow velocities (16 cm ⋅ min^–1^) indicates a differential flow response in EPS production. The high biomass but comparatively low DNA content achieved under moderate flow indicates an enhanced proportion of extracellular components in these biofilms. In accordance with this observation, previous studies have demonstrated how shear stress fosters adhesion and structural integrity of biofilms by stimulating EPS secretion (Hou et al., 2018; Liu and Tay, 2002). A molecular basis for these observations is given by Rodesney et al. (2017), who identified mechanosensory elements in *P. aeruginosa* that respond to applied shear forces by activating cyclic-di-GMP signaling pathways known to stimulate biofilm formation and EPS production (Liu et al., 2022; Simm et al., 2004; Steiner et al., 2012; Valentini and Filloux, 2016). Moreover, previous studies concluded that an impaired nutrient availability under low shear stress constrains EPS production, resulting in fragile biofilms prone to detachment (Nilsson et al., 2025; Wang et al., 2020). This supports our observations of greater biofilm rigidity under moderate compared to low flow conditions. It has to be acknowledged that direct visualization of biofilm architecture, thickness and EPS distribution by Confocal Laser Scanning Microscopy (CLSM) would complement these functional measurements. However, CLSM analysis of biofilms on rough titanium surfaces presents known technical challenges: The optical properties of the (structured) metallic substrate, particularly opacity and reflection/scattering, limit axial resolution and complicate the localization of the biofilm-substrate interface (Hirai et al., 2025; Kleine et al., 2019). These constraints were confirmed in our preliminary experiments and necessitated the quantitative approach employed here. In future studies we plan to overcome these limitations by complementary techniques such as scanning ion conductance microscopy (SICM) for topographical analysis on opaque substrates, as suggested by Hirai et al. (2025).

The use of qPCR, paired with the *de novo* development of primers to target the single-copy *rpoB* gene, allowed us to examine the precise species distribution at varying hydrodynamic conditions. This approach was chosen since the single-copy target allows for a direct translation of quantified DNA to cell abundance and thus maintains comparability between the different species. In contrast to the remaining strains, *A. naeslundii*, *V. parvula* and *A. actinomycetemcomitans* form highly persistent multicellular aggregates, even in planktonic cultures, making a comparison based on CFU not feasible or at least heavily biased, as described previously (Harmsen et al., 2000; Wagner et al., 1993).

Resulting species abundances in our reference biofilms grown under static conditions showed a generally comparable community structure to the static model described by Sánchez et al. (2011), using identical species but on hydroxyapatite (HA) surfaces. The main difference constituted the higher relative abundance of the early colonizers S. *oralis* and *A. naeslundii* in our model (comparing equivalent cultivation periods). This could be attributed to the generally higher CFU concentrations in our inoculum, or to surface-dependent colonization dynamics, whereby early colonizers may colonize the rough titanium surfaces more successfully than the smoother HA. Different DNA-based species quantification techniques used in both studies (qPCR vs. T-RFLP) might also introduce a methodological bias. In particular, *A. naeslundii* is known to resist standard DNA extraction protocols (Barsotti et al., 1987; Li et al., 2020), an issue we addressed in our extraction approach, potentially accounting for its higher abundance in our model. This species-specific extraction bias represents an important methodological consideration for any multi-species biofilm study, when assessing species abundances. Several studies have shown that extraction efficiencies across methods can vary substantially between species, particularly with extraction methods that rely on commercial kits, resulting in altered community compositions (Abusleme et al., 2014; Corcoll et al., 2017; Li et al., 2020). Our own experiments revealed that standard DNA extraction kits produced highly variable recovery across our six species (data not shown). To minimize this bias, we employed a phenol-chloroform based protocol, known to significantly increase DNA recovery (Rosenbaum et al., 2019), combined this with an extensive multi-step lysis to equalize efficiency across species as far as possible. While residual extraction bias cannot be entirely excluded, our data presents a strong correlation of biomass measurements and DNA quantification, as well as consistency with published community compositions. Researchers adapting our system for quantitative multi-species studies should therefore carefully select and optimize the DNA extraction method for their target species.

The introduction of flow resulted in a distinct shift in the biofilm community composition within our model. Lower flow conditions (8 cm ⋅ min^–1^) promoted middle-colonizer dominance, characterized by a substantial over-representation of *F. nucleatum* (> 60%). This predominance aligns with previous observations of *in vitro* and *in vivo* studies: Guggenheim et al. (2001) reported *F. nucleatum* accounting for more than 50% of the total cell load in their static model, while Moore and Moore (1994) identified it as one of the most prevalent isolates from gingival-crevice plaque. More recently, *F. nucleatum* was identified as the most abundant species which, alongside *Veillonella* spp., forms part of the shared core microbiome of healthy and diseased peri-implant sites (Belibasakis and Manoil, 2021; Chen et al., 2022).

The elevated abundance of late colonizers observed under lower flow conditions in our model (*P. gingivalis*: ∼5%; *A. actinomycetemcomitans*: ∼8%) is potentially facilitated by the established function of *F. nucleatum* as a bridging organism. Adhering to both early and late colonizers, *F. nucleatum* enables coaggregation and mutualistic relationships that promote pathogen integration into developing biofilms (Chen et al., 2022). The substantial *F. nucleatum* presence under low shear exposure likely provided attachment and integration for late colonizer establishment in our model.

The progressive reduction of late colonizers with increasing shear observed in our study can be attributed to multiple factors. A structural analysis of a static 10-species subgingival Zurich biofilm model (Ammann et al., 2012) by fluorescence *in situ* hybridization (FISH) localized the pathogenic late colonizers, including *P. gingivalis*, loosely attached in the top layer of biofilms. This spatial distribution renders them particularly vulnerable to detachment under elevated hydrodynamic forces. Additionally, late colonizer establishment requires potentially extended maturation periods: Sánchez et al. (2011) observed low relative abundances for *P. gingivalis* during initial biofilm development in their static model, reaching peak abundance only after 144 h of cultivation. A cultivation period of 84 h, as in our flow-exposed biofilm model, may have been insufficient for late colonizers to achieve substantial representation under the additional selective pressure of elevated shear stress. In general, a low pathogen abundance is entirely common for *in vivo* biofilms on dental implants: A systematic analysis of implant infections by Jia et al. (2024) reported relative abundances of *Porphyromonas* spp. between 6.1% under peri-implantitis and 2.6% under peri-implant mucositis conditions. These proportions align with the keystone pathogen hypothesis, stating that low-abundance pathogens can orchestrate inflammatory diseases by transforming commensal microbial communities into dysbiosis (Hajishengallis et al., 2012). The flow of gingival crevicular fluid (GCF) is closely correlated with the severity of periodontal inflammation, serving as a direct indicator of tissue response; it increases markedly due to enhanced vascular permeability and epithelial ulceration at inflamed sites. While healthy gingiva produces minimal GCF, inflammation leads to a substantial rise in both volume and compositional complexity, transforming it into an exudate characteristic of inflammatory processes (Barros et al., 2016).

The balanced microbial community we observed under moderate flow conditions (16 cm ⋅ min^–1^), with early and middle colonizers exhibiting comparable abundances, likely reflects an optimal balance between hydrodynamic forces and bacterial colonization dynamics. As described by Beyenal and Lewandowski (2002), enhanced flow velocities increase the mass transfer, allowing nutrient transport also into deeper biofilm layers. This more uniform nutrient distribution could support the maintenance of early colonizer populations alongside middle colonizers. They further describe how flow velocities beyond a certain threshold reinforce denser biofilm structures to resist shear stress at the expense of internal nutrient transfer, potentially explaining the recurrent suppression of early colonizers under higher flow conditions (32 cm ⋅ min^–1^). The reduced diversity under high flow aligns with previous findings that high shear stress reduces biofilm diversity and slows maturation (Rochex et al., 2008). Across all flow rates, the balanced community observed under moderate flow corresponds the closest to *in vivo* peri-implant microbial profiles: In clinical biofilm compositions reported by Jia et al. (2024), the genera *Streptococcus* and *Fusobacterium* exhibited the highest abundances of comparable magnitude, while also *Actinomyces* and *Veillonella* presented slightly lower yet relatively similar ratios. However, a direct comparison with *in vivo* situations remains challenging, due to substantially higher microbial complexity and diversity, infection stage-dependent variations and highly patient-specific compositions (Dieckow et al., 2024; Jia et al., 2024).

We assessed the structural integrity of flow-exposed biofilms by applying mechanical stress in the form of standardized washing steps. This revealed the presence of a persistent basal layer within all biofilms, presenting similar DNA content across all flow rates but significantly enhanced total biomass at moderate flow conditions (16 cm ⋅ min^–1^). These results suggest that shear exposure up to a certain threshold not only promotes EPS production but also strengthens the cohesive properties of the extracellular matrix. Consistent with these findings, Gloag et al. (2020) described how shear stress enhances both rigidity and also elasticity of biofilms, proposing an improved interaction between EPS compounds as a shear response. Our observation of a resilient basal biofilm layer has likewise been documented by Simões et al. (2022) for biofilms of *P. fluorescens* formed under various shear stress conditions. They demonstrated that even after combined mechanical and chemical treatment, a firmly adherent basal layer of predominantly viable cells was retained on the surface, whereby biofilm resilience increased proportionally with shear exposure during formation.

While the mechanical resistance of basal biofilm layers has been well described for environmental or industrial settings (Lemos et al., 2015; Möhle et al., 2007; Paul et al., 2012; Simões et al., 2022), its compositional stability in oral biofilms has received limited attention. The relative species distribution in the basal layer of our model biofilms remained largely uniform across all flow rates. Characterized by a clear middle colonizer dominance, the basal layer showed flow-dependent changes mainly in the progressive reduction of late colonizers with increasing flow, as also observed for untreated biofilms. This suggests that the species composition of this foundational community is less influenced by shear-induced selection, but rather by initial surface colonization dynamics. The presence of middle colonizers such as *F. nucleatum* directly at the substrate surface has been previously observed by Ammann et al. (2012), who described a stratified structure within their static 10-species subgingival biofilm model. In connection with peri-implant treatment strategies, observing such a resilient basal layer is highly relevant, as it could serve as a persistent reservoir for biofilm reformation after debridement.

Our system successfully demonstrates the production of representative oral biofilms, robust enough to endure potential treatment evaluations. The creation of more resilient *in vitro* biofilms through adapted flow exposure also facilitates a more realistic assessment of treatment efficacy: Longer maturation periods and higher diversity with numerous interspecies interactions grant *in vivo* biofilms cross-protections against antimicrobials such as hydrogen peroxide (Dieckow et al., 2024), which can hardly be recreated by static models. Notably, our system achieved stable multi-species biofilms without requiring pooled human saliva, which is used in most oral biofilm models (Darrene and Cecile, 2016), despite its inherent donor-dependent inter-individual variability (Quintana et al., 2009).

While our model enables valuable insights into flow-dependent variations of biofilm development, mechanical properties and microbial communities, the standard qPCR analysis has potential inherent limitations. Quantifying the total DNA content, including non-viable cells, does not allow for a conclusion about the proportion of metabolically active cells. We explored several viability assessment approaches and identified technical challenges, arising from the anaerobic nature of our samples, that should be considered by researchers planning to adapt this system: Without a suitable anaerobic workstation, the exposure of biofilms to oxygen during sampling and processing compromises standard viability measurements. Obligate anaerobic species potentially perish during handling of metabolic activity assays (e.g., tetrazolium reduction) or CFU enumeration. Conventional Live/Dead fluorescent staining faces the additional issue that the sudden availability of oxygen as electron acceptor can elevate the membrane potential of viable facultative anaerobes enough to take up propidium ions (“dead stain”) through intact membranes (Kirchhoff and Cypionka, 2017). Future studies will address this by utilizing an anaerobic workstation to perform PMA-qPCR (“viability” qPCR), as well as species localization by FISH and CLSM. The decoupled flow control of our system will also permit to challenge biofilms with controlled shear pulses or flow ramps, without impairing culture stability. Other potential applications include sequential sampling of biofilms at multiple timepoints to investigate flow-dependant colonization kinetics and species succession, as well as the customization of flow conditions to resemble the dynamic flow changes in the oral cavity throughout the day. This could even improve biofilm formation, as Tsagkari et al. (2022) demonstrated that non-uniform flow patterns can enhance biomass accumulation.

Overall, our dual-cycle approach provides a versatile platform, with the ability to independently control flow exposure and cultivation conditions. This approach addresses specific limitations experienced with existing dynamic biofilm systems while expanding experimental flexibility: The CDC biofilm reactor employs rotational stirring to create a flow that is characterized by unsteady turbulent structures and produces a non-uniform shear exposure, whereby exact flow characterization requires computational fluid dynamics modeling (Johnson et al., 2021). In comparison to this, our MRD configuration provides uniform, quantifiable flow across all sample surfaces, with a gentle tilting motion to prevent gas and debris accumulation while preserving laminar flow characteristics. Traditional in-line MRD-chemostat configurations couple the flow rate through the biofilm device directly to the culture efflux (Blanc et al., 2014; Jass et al., 1995), creating a fixed relationship between biofilm shear exposure and culture turnover rate. This coupling restricts experimental flexibility, since the flow through cannot be fully regulated without altering steady-state culture conditions. Maintaining steady-state culture conditions, however, is critical for physiological relevance, as these conditions are required for bacteria to express cell wall proteins and respond to environmental stimuli similarly to *in vivo* cells, as opposed to batch-grown cultures (Drake and Brogden, 2014). The parallel recirculating design presented here eliminates this trade-off, enabling independent modulation of both parameters across wider experimental ranges. This parametric independence also makes high-shear studies feasible: An in-line system, connected to either a chemostat or a buffered batch culture, which operates at the flow velocities examined here (8–32 cm⋅min^–1^) would consume 50–80 L of medium/culture over 84 h, compared to ∼4 L with our recirculating configuration where medium consumption is independent of the MRD flow velocity. The low media consumption of our system is also supported by the small-scale reactor volume (50 mL culture volume compared, e.g., to 350 mL minimum working volume for CDC reactors) that further reduces the required medium feed to maintain steady-state conditions for fastidious anaerobes. This efficiency is particularly important for studies that require costly culture media supplements. Beyond this resource efficiency, the dual-loop design provides new experimental degrees of freedom that were impractical with previous systems. In future studies, multiple MRDs can be connected in parallel to a single chemostat (using separate pumps), allowing the simultaneous examination of different flow conditions while excluding variability from different culture conditions that might occur across independent experimental runs. These capabilities make this configuration readily adaptable to various scenarios. Culture conditions can be altered by nutrient addition or limitation to mimic specific stages of infections or inflammatory responses. The system provides a generalizable tool to simulate diverse biomaterial-associated infections, including urinary catheters, orthopedic implants, vascular grafts or prosthetic heart valves.

## Data Availability

The raw data supporting the conclusions of this article will be made available by the authors, without undue reservation.
