# Supplementary material for: A dual-loop chemostat to investigate multi-species biofilms on implant surfaces under adjustable flow conditions
Source: Front Microbiol. 2026 Feb 13;17:1751315. doi: 10.3389/fmicb.2026.1751315 (PMC12946110; doi:10.3389/fmicb.2026.1751315)
Supplement: Supplementary file 1 [file Data_Sheet_1.pdf]

Supplementary Material

1

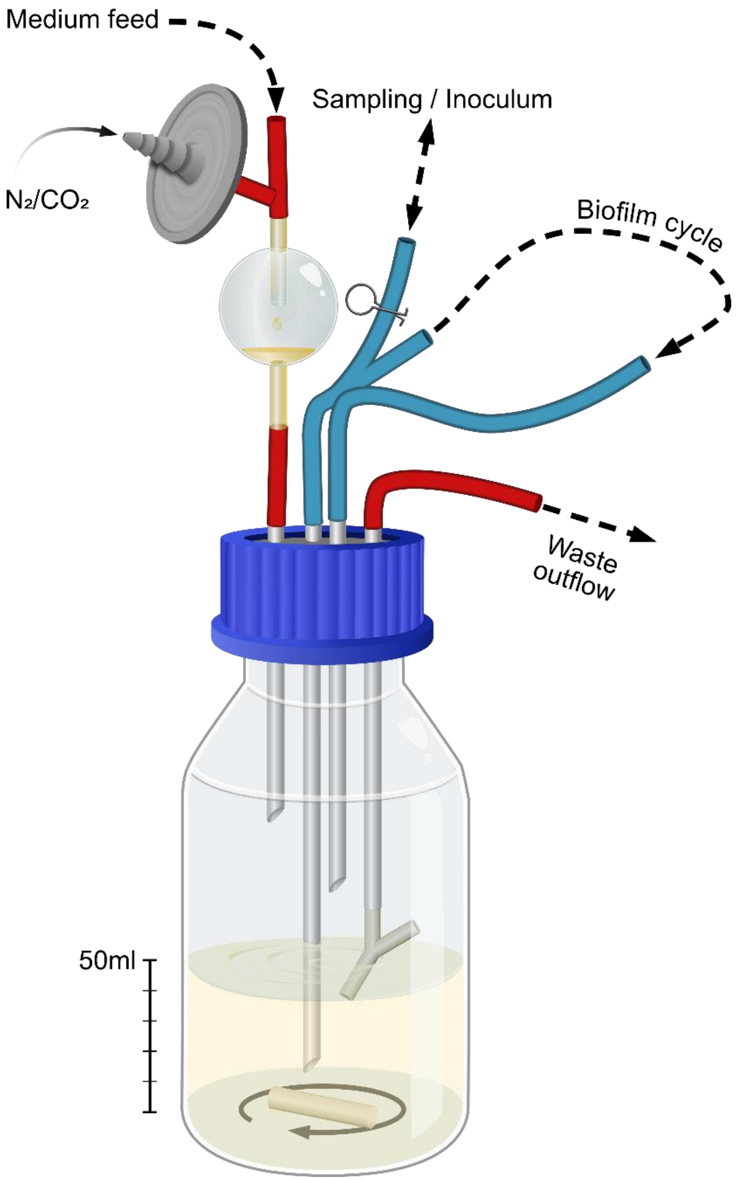

Supplementary Figure 1: Bioreactor setup – Schematic illustration.
Detailed display, illustrating the small-scale reactor vessel designed for the *in vitro* model.
Red tubing represents the cultivation cycle, blue tubing the flow-controlled biofilm formation cycle.

Supplementary Table 1: Statistical analysis for normal distribution of datapoints within all groups.

Biofilm Analysis	Flow Velocities	Treatment	n	W- statistic	z-statistic	p-value	Distribution
Biomass CV-assay	8 cm/min	Untreated	7	0.716	2.544	0.006	**Non-normal**
	16cm/min		7	0.926	-0.049	0.520	Normal
	32cm/min		7	0.901	0.413	0.340	Normal
	8 cm/min	Washed	6	0.991	-2.396	0.992	Normal
	16cm/min		5	0.909	0.097	0.461	Normal
	32cm/min		5	0.815	1.241	0.107	Normal
Total DNA	8 cm/min	Untreated	4	0.966	-0.902	0.817	Normal
	16cm/min		4	0.957	-0.696	0.767	Normal
	32cm/min		4	0.895	0.235	0.407	Normal
	8 cm/min	Washed	3	0.873	0.510	0.305	Normal
	16cm/min		3	0.999	-2.441	0.993	Normal
	32cm/min		3	0.890	0.377	0.353	Normal

Shapiro-Wilk test for normality distribution.
Distribution assessment: Normal ($p > 0.05$), Non-normal ($p < 0.05$).

Supplementary Table 2: Statistical analysis of untreated biofilm biomass (CV) at different flow velocities.

Compared Flow Velocities	Treatment	Rank Sum (Group1)	Rank Sum (Group2)	z-statistic	p-value	Significance
**8** vs **16 cm/min**	Untreated	42	63	-1.342	0.209	ns
**8** vs **32 cm/min**		60	45	0.958	0.383	ns
**16** vs **32 cm/min**		77	28	3.130	0.0006	***

Untreated samples: Kruskal-Wallis test: $x^2(2) = 8.38$, $p = 0.0152$
Mann-Whitney U test (two-tailed) with exact p-values for small sample sizes
Significance: *** $p < 0.001$, ** $p < 0.01$, * $p < 0.05$, ns = not significant

Supplementary Table 3: Statistical analysis of washed biofilm biomass (CV) at different flow velocities.

Compared Flow Velocities	Treatment	Mean Difference	SEM	t-statistic	df	p-value	Significance
**8** vs **16 cm/min**	Washed	-0.163	0.055	-2.96	9	0.016	*
**8** vs **32 cm/min**		0.103	0.049	2.09	9	0.066	ns
**16** vs **32 cm/min**		0.266	0.054	4.89	8	0.001	**

Washed samples: One-way ANOVA: $F(2,13) = 12.15$, $p < 0.001$.
Two-sample t-tests with equal variance assumed.
SEM = Standard Error of Mean.
df = Degrees of Freedom.
Significance: *** $p < 0.001$, ** $p < 0.01$, * $p < 0.05$, ns = not significant

Supplementary Table 4: Statistical analysis of total biofilm DNA at different flow velocities.

Compared Flow Velocities	Treatment	Mean Difference (µg)	SEM	t-statistic	df	p-value	Significance
**8** vs **16 cm/min**		19.3	6.24	3.09	6	0.021	*
**8** vs **32 cm/min**	Untreated	23.4	5.57	-4.19	6	0.006	**
**16** vs **32 cm/min**		4.1	5.04	0.81	6	0.448	ns
**8** vs **16 cm/min**		1.5	1.10	1.36	4	0.246	ns
**8** vs **32 cm/min**	Washed	1.2	1.40	-0.83	4	0.452	ns
**16** vs **32 cm/min**		-0.3	0.97	-0.34	4	0.750	ns

Untreated samples: One-way ANOVA: $F(2,9) = 9.79$, $p = 0.006$
Washed samples: One-way ANOVA: $F(2,6) = 0.90$, $p = 0.454$
Two-sample t-tests with equal variance assumed.
SEM = Standard Error of Mean.
df = Degrees of Freedom.
Significance: *** $p < 0.001$, ** $p < 0.01$, * $p < 0.05$, ns = not significant

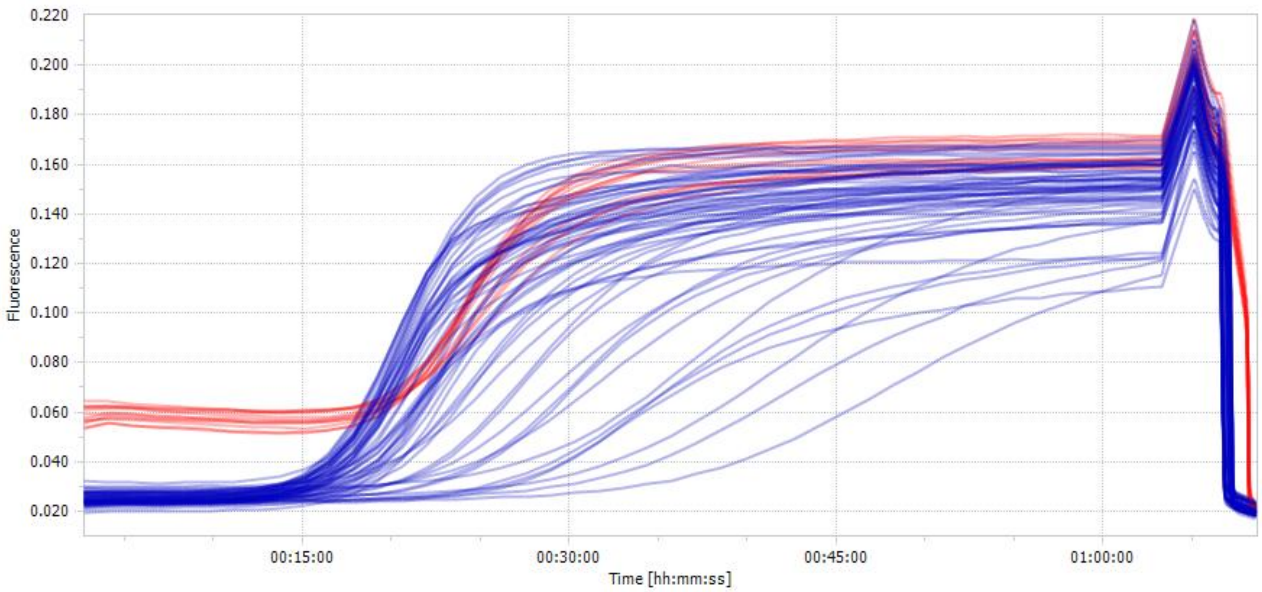

Supplementary Figure 2: Elevated fluorescence baseline of *A. naeslundii* DNA. Raw data of fluorescence curves, exported from the LightCycler® 96 Application Software (V1.1, Roche Diagnostics). A gradient qPCR was carried out for all six primer-pars, assessing annealing temperatures between 62°C and 69°C. DNA concentration in all samples was 10 ng per well. The graph reveals the elevated initial baseline fluorescence of *A. naeslundii* DNA before the beginning of amplification. All samples of *A. naeslundii* DNA presented a baseline fluorescence between 0.055 and 0.065, whereas all DNA samples of the five remaining strains had a baseline fluorescence between 0.030 and 0.020.

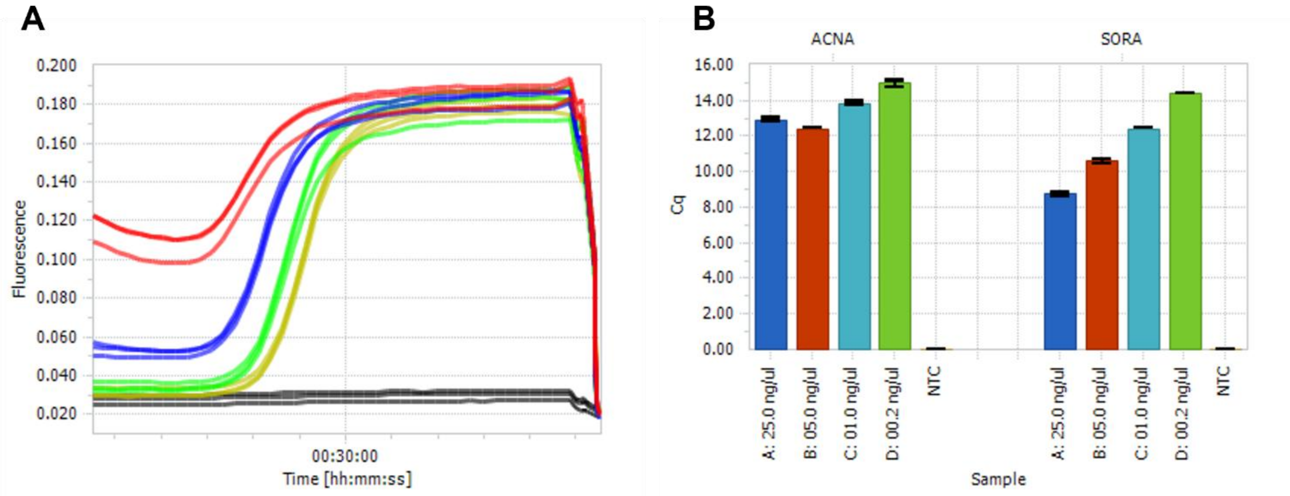

Supplementary Figure 3: Concentration-dependent DNA fluorescence of *A. naeslundii*. **A:** Raw data of fluorescence curves, exported from the LightCycler® 96 Application Software (V1.1, Roche Diagnostics). qPCR was carried out in triplicates with *A. naeslundii* DNA of different concentrations in a 5-fold dilution series. At the highest template concentration of 25 ng/µl (red) the baseline fluorescence was elevated to ~0.120 and decreased to ~0.050 at a dilution to 5 ng/µl (blue). Sample DNA concentrations of 1 ng/µl (green) and 0.2 ng/µl (yellow) resulted in an initial baseline fluorescence comparable to those of the remaining strains, with values between 0.04 and 0.06. **B:** C_q values of 5-fold dilutions of *A. naeslundii* DNA (ACNA) and *S. oralis* DNA (SORA) as reference. Compared to the reference, the C_q values for A. naeslundii DNA, especially the high concentrations, were significantly enhanced, while also presenting an impaled reproducibility between the replicates. The 25 ng/µL samples even yielded higher C_q values than the 5 ng/µL samples, which produced an incorrect concentration assignment of the qPCR results.

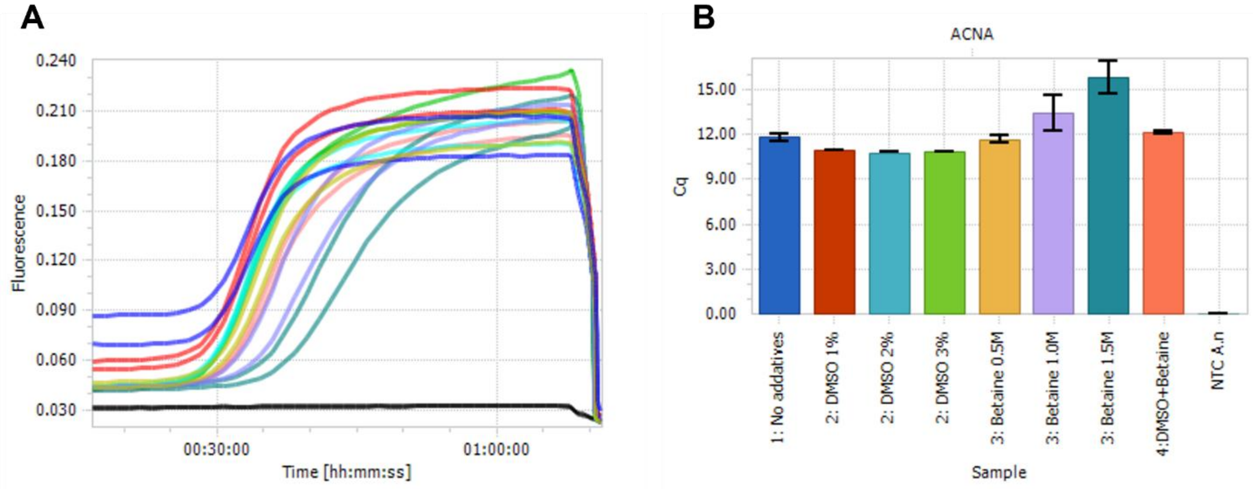

Supplementary Figure 4: Influence of betaine and DMSO on qPCR performance. **A:** Raw data of fluorescence curves, exported from the LightCycler® 96 Application Software (V1.1, Roche Diagnostics). qPCR amplification curves of *A. naeslundii* DNA (25ng/µl) after prolonged preincubation (15min, 95°C) with MasterMix variations: No additives (blue), 1% DMSO (red), 2% DMSO (turquoise), 3% DMSO (light-green), 0.5M Betaine (yellow), 1M Betaine (purple), 1.5M Betaine (dark-green), 1% DMSO + 0.5M Betaine (orange) and no template control (black). Initial fluorescence baseline decreased slightly with no additives but longer preincubation, while still showing poor consistency of replicates. The addition of 1% DMSO reduced the baseline fluorescence further and also enhanced reproducibility. At a concentration of 2% DMSO, the baseline fluorescence reached values close to 0.04 and thus a niveau that corresponds to reference measurements of the other strains. Betaine addition in any tested concentration reduced baseline fluorescence back to the reference standard, but also delayed template amplification, becoming more pronounced with increasing concentrations.

B: C_q values, achieved by replicates *A. naeslundii* DNA (ACNA) for qPCR measurements with various additives in the MasterMix. Average reference C_q of samples without any additives was at 11.81. Addition of 2% DMSO reduced the C_q to the lowest value (C_q=10.77), concurrently achieving the best reproducibility. The presence of betaine, although successfully reducing baseline fluorescence, enhanced the C_q again and further compromised reproducibility. Hence, the addition of 2% DMSO was chosen to be the optimum to counteract the increased baseline issue for *A. naeslundii* DNA.

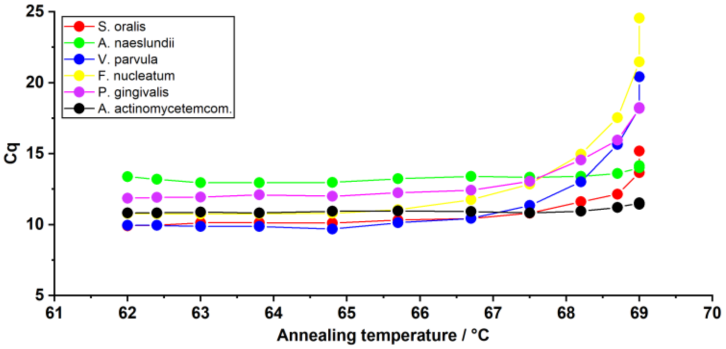

Supplementary Figure 5: Evaluation of optimal annealing temperature. Quantification cycle (C_q) is represented as a function of annealing temperature during a gradient qPCR, carried out for all six primer-pars. Annealing temperatures between 62°C and 69°C were assessed. DNA concentration in all samples was 10 ng per well. The ideal condition, combining specific annealing, absence of dimers and uninhibited amplification, was determined at an optimal annealing temperature of 66°C.

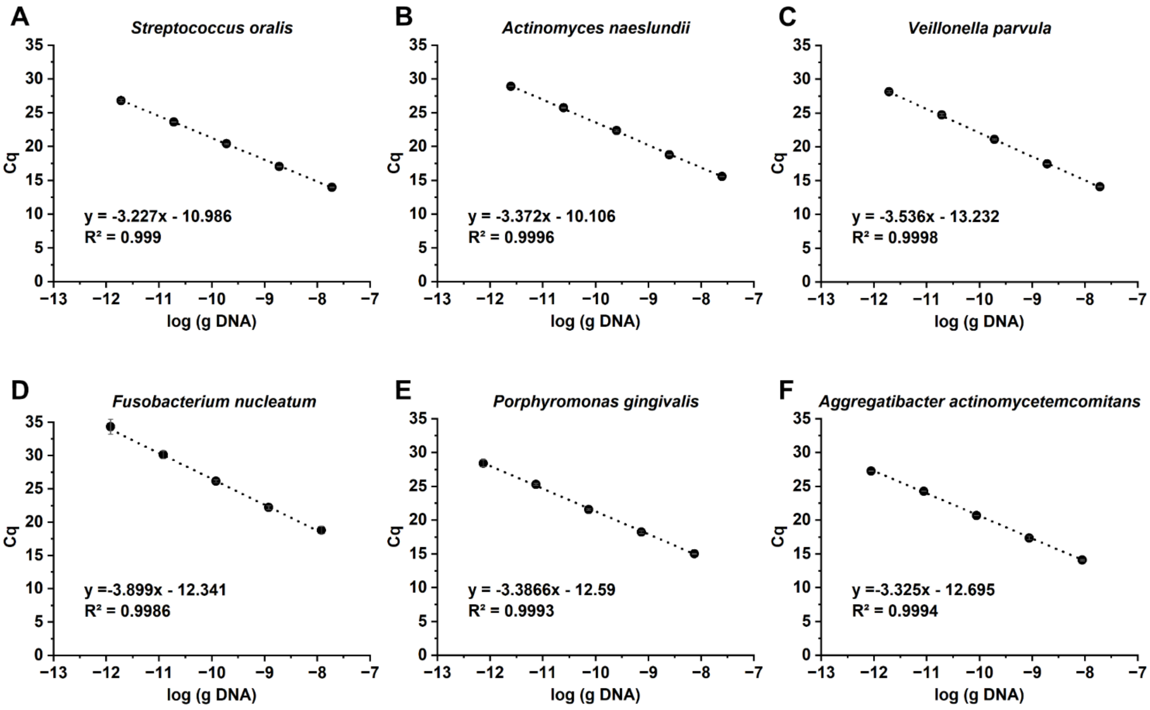

Supplementary Figure 6: Standard curves for primer performance evaluation. Graphical appearance of logarithmic DNA amounts, plotted against their corresponding mean C_q values, determined by SYBR-based qPCR in duplicates. Known concentrations of DNA were serially diluted to obtain a 10-fold range between 10^{-8} to 10^{-12} g DNA. Standard curves were generated respectively by linear regression analysis, allowing for calculation of amplification efficiencies (shown in table 2). DNA was extracted from axenic cultures of **(A)** *S. oralis*, **(B)** *A. naeslundii*, **(C)** *V. parvula*, **(D)** *F. nucleatum*, **(E)** *P. gingivalis* and **(F)** *A. actinomycetemcomitans*. Extrapolation from these standard curves enables to determine the DNA amount for each species from the C_q values of unknown (mixed) samples.

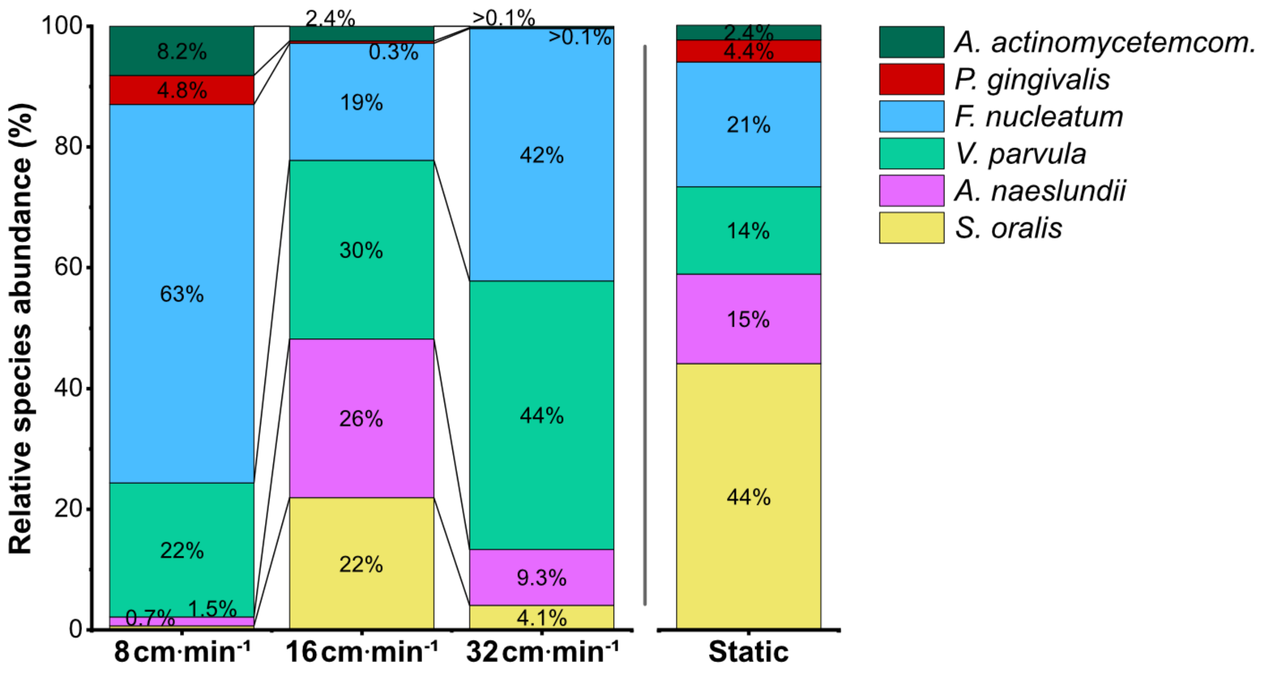

Supplementary Figure 7: Microbial community composition: Dynamic vs. static biofilm model. Relative species abundance (%) in untreated biofilms cultivated under varying flow velocities compared to biofilms obtained under static conditions. Six-species biofilms were grown for 84 h on Ti-stubs/-discs, either in a MRD under varying flow velocities (dynamic model) or within the wells of a 24-well tissue culture plate (static model). Species quantification of biofilm DNA was performed by SYBR-based qPCR with species-specific primers targeting the single-copy *rpoB* gene.

Flow-rate estimation from perfusion measurements given by Krastev & Filipov (2020)

Blood perfusion values reported in the cited laser Doppler flowmetry studies (expressed in $mV\cdot min^{-1}\cdot 100\ g^{-1}$) were used as input to estimate system-specific volumetric flow rates. Briefly, perfusion signals were first normalized to the baseline condition and then converted to mean flow velocity by applying the Bernoulli equation under the simplifying assumptions of steady, laminar flow of a Newtonian, incompressible fluid and negligible elevation changes:

$$\Delta P = \frac{1}{2}\rho(\nu^2 - v_0^2)$$

where ΔP is the assumed pressure difference driving flow, ρ is fluid density, andν^2 and v_0^2 are the mean velocities under inflammatory and baseline conditions, respectively. Volumetric flow rate was subsequently calculated as Q = vA, where A is the effective cross-sectional area of the flow domain used in the experimental flow model device. The resulting flow rates **should be interpreted as first-order estimates** intended to reproduce relative changes in peri-implant microcirculation reported in the literature, rather than absolute physiological blood flow values.
